# In-hospital neonatal maternal separation as early-life stress and neurodevelopmental disorders: a cross-sectional and population-based study

**DOI:** 10.3389/fpsyt.2025.1572600

**Published:** 2025-06-27

**Authors:** Quan Zhou, Senrui Liang, Yanyan Sun, Jun Fan, Zaisheng Qin

**Affiliations:** ^1^ Department of Anesthesiology, Nanfang Hospital, Southern Medical University, Guangzhou, China; ^2^ Guangdong-Hong Kong-Macao Greater Bay Area Center for Brain Science and Brain-Inspired Intelligence, Southern Medical University, Guangzhou, China; ^3^ Department of Anesthesiology, Shenzhen University General Hospital, Shenzhen University, Shenzhen, China; ^4^ Department of Anesthesiology, Guangzhou Women and Children’s Medical Center, Guangzhou Medical University, Guangdong Provincial Clinical Research Center for Child Health, Guangzhou, China

**Keywords:** neonatal maternal separation, neurodevelopment disorders, in-hospital, early-life stress, children and adolescents

## Abstract

**Background:**

Neurodevelopmental disorders are the most common psychiatric disorders in children and adolescents; however, preventing their onset and progression remains challenging. Due to ethical constraints, population-based studies investigating neonatal maternal separation (NMS) as an early-life stress in children and adolescents are scarce.

**Methods:**

We analyzed data from five cycles (1999–2000 to 2007–2008) of the National Health and Nutrition Examination Survey, focusing on participants aged 1–15 years. The participants were identified using their replies to survey interview questions. The exposure of interest was in-hospital NMS, while the primary outcome was neurodevelopmental disorders, which include attention deficit hyperactivity disorder, learning disability, and special education or early intervention services. Multifactorial weighted logistic regressions with confounder adjustment were performed for participants with available data on the exposures, confounders, and outcome.

**Results:**

Overall, data from 15502 participants (mean age, 8.05 years [SE 0.06]; 7759 males [weighted, 50.99%]) were analyzed. Neurodevelopmental disorders were more common in participants with prior experience of in-hospital NMS. Multifactorial weighted logistic regression model analyses revealed a significant positive association between in-hospital NMS and the occurrence risk of neurodevelopmental disorders (adjusted OR 1.82 [95% CI 1.40–2.37], P < 0.001). The pattern of association was largely consistent across the subgroups.

**Conclusions:**

In-hospital NMS as an early-life stress is associated with an increased incidence of neurodevelopmental disorders in children and adolescents. This may be a targetable risk factor for future trials to examine the long-term outcomes of newborn-mother connection interventions and to tailor preventative and treatment interventions.

## 1 Introduction

Neurodevelopmental disorders are complex and heterogeneous disorders characterized by developmental deficits resulting in learning, memory, attention, and social interaction impairments (1). They frequently occur concurrently and are often co-diagnosed with other physical or mental health disorders (1, 2). Neurodevelopmental disorders have a significant socio-economic impact and are the most common psychiatric and behavioral disorders in children and adolescents (2, 3).

Although several studies have shown that maternal separation, as a form of early-life stress, is linked to neurodevelopmental disorders (4), available population studies focused primarily on the causes of maternal separation, including early institutionalization, war, separation during asylum, trafficking, and abandonment when parents leave for economic or other reasons (5, 6). Neonatal maternal separation (NMS) is an important but often overlooked form of maternal separation and typically refers to the behavior of a newborn who is being separated from its mother immediately or for an early period after birth, mostly occurring in hospitals, especially in special neonatal care facilities or neonatal intensive care units (7, 8). NMS also occurs during epidemics of some infectious diseases, such as tuberculosis and COVID-19 (9–13). The period when a newborn is separated from its mother is critical for the infant’s psychophysiological development and sensitivity, which can reprogram future physiology and behavior (7). Studies have shown the benefits of maintaining close contact between a mother and an infant immediately after birth (14). Other studies have shown that changes in hospital nursing care models (e.g., family integrated care and kangaroo mother care) increased the opportunities for newborns to interact with their parents (15, 16). Increasing evidence also suggests that NMS may be a risk factor for neurodevelopmental disorders due to deleterious epigenetic regulation caused by separation stress in animal models (7). However, owing to ethical concerns, few clinical studies have focused on in-hospital NMS and its long-term outcomes in children and adolescents.

To our knowledge, only one cohort, the Finnish Christmas Seal Home, which was temporarily separated from its family and placed in appropriate care facilities immediately after birth for an average of 7 months due to a family history of tuberculosis between 1945 and 1965, examined the long-term outcomes of NMS (10–13). The research team examined the effects of NMS on schizophrenia and found that separation at birth was not linked to schizophrenia or other psychiatric disorders (10). However, further analyses revealed that NMS had an impact on later criminality of the offspring, hospitalization for depression in adulthood, and substance use disorders (11–13). Currently, no cross-sectional study has used a representative national sample to investigate the effects of in-hospital NMS. Consequently, there is limited knowledge regarding whether in-hospital NMS is a potential risk factor for neurodevelopmental disorders in children and adolescents.

To address this gap, we utilized data from the National Health and Nutrition Examination Survey (NHANES), a prospective cohort of nationally representative samples in the United States, to examine the association between in-hospital NMS and neurodevelopmental disorders in children and adolescents, controlling for demographic variables and early life factors. We hypothesized that in-hospital NMS would be associated with a higher risk of neurodevelopmental disorders.

## 2 Methods

### 2.1 Study design and participants

NHANES used a complex, stratified, multistage probability cluster sampling and cross-sectional design to collect health and nutrition data from a representative sample of the civilian noninstitutionalized population of the United States of America. Participants are selected every 2 years since 1999 to complete an in-person, structured interview at home and to undergo standardized laboratory examinations and laboratory tests in mobile health centers. NHANES questionnaires cover several topics, including demographic characteristics, lifestyle, general health, and medical history (17). The study protocols for NHANES were approved by the National Center for Health Statistics Ethics Review Board. All adult participants provided written informed consent. The deidentified data analyzed in this study were publicly available at NHANES; therefore, the study did not require review board approval. This study followed the Strengthening the Reporting of Observational Studies in Epidemiology reporting guidelines for cohort studies.

**Figure 1:**
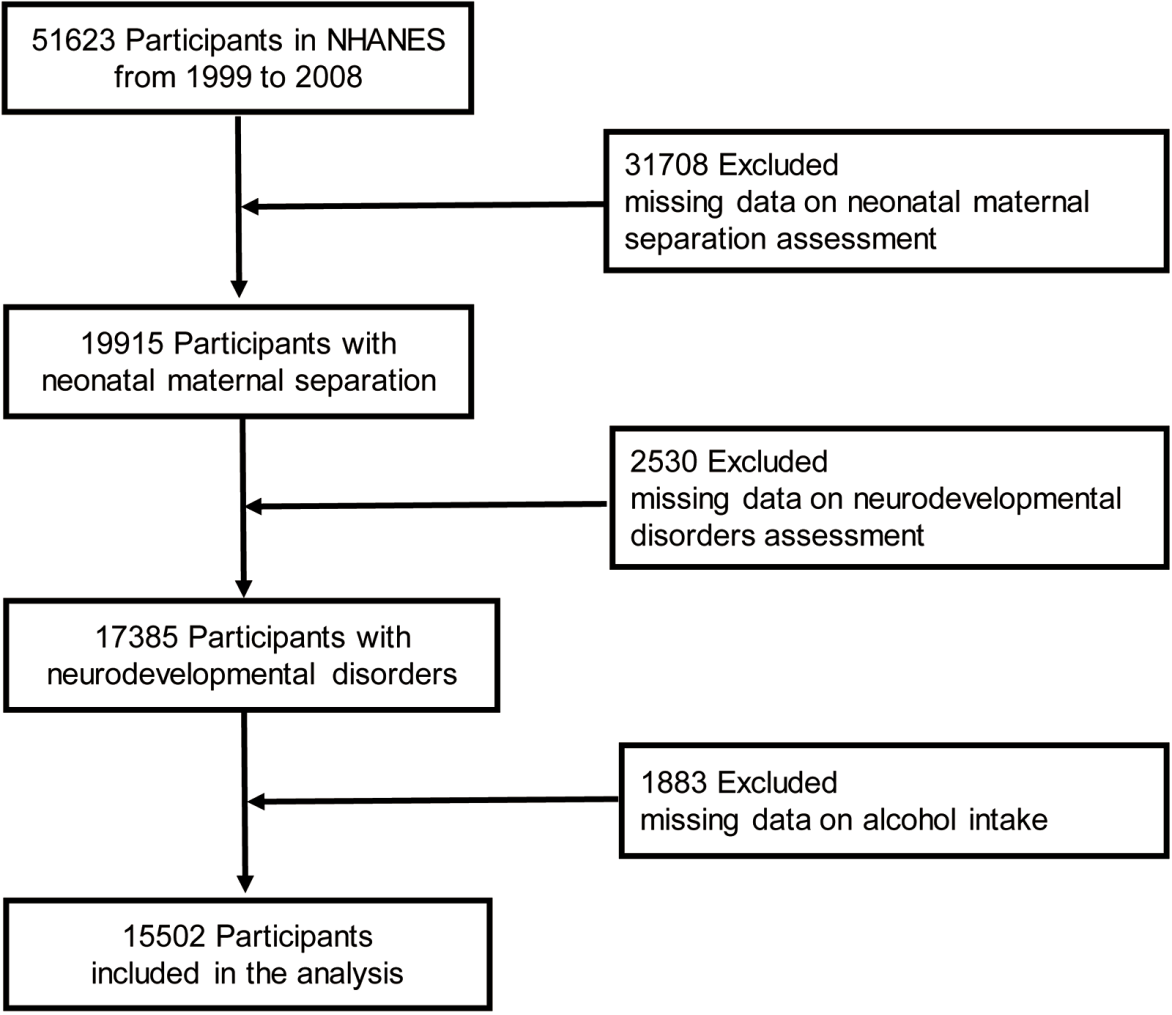
Study flowchart.

Children and adolescents <15 years of age who participated in five continuous NHANES survey cycles (1999–2000 to 2007–2008) and who had available data on in-hospital NMS and neurodevelopmental disorder questionnaires were included in the analysis. Participants for whom data on the intake of alcohol and individual foods (first day) were missing were further excluded because weights for complicated sampling designs were created using the sampling weight of the first-day dietary data according to NHANES analytical guidelines (18). **Figure 1** shows the participant engagement flowchart.

### 2.2 Exposure and outcome assessment

Exposure and outcome assessment were based on responses from parents, guardians, or other adult guardians. In-hospital NMS was assumed as a positive answer to the question, “Did [Sample Person (SP) name] receive any newborn care in an intensive care unit, premature nursery, or any other type of special care facility?” Such settings often necessitate the physical separation of mothers and their newborns, especially in cases of preterm birth or common neonatal illness, limiting maternal presence and direct contact, resulting in maternal-infant separation (19–21).

The primary outcome, neurodevelopmental disorders, was defined by a response of “Yes” to one of the three questions as described previously (22): “Has a representative from a school or a health professional ever told you/SP that s/he/SP had a learning disability?” “Has a doctor or health professional ever told you/SP that you/s/he/SP had attention deficit disorder?” or “Does SP receive special education or early intervention services?”

### 2.3 Covariates

The selection of confounders was based on existing studies and theoretical assumptions. All confounders included were routinely associated with NMS and neurodevelopmental disorders. Population baseline data included sex, age, body mass index (BMI), race, family income to poverty ratio (PIR), health insurance status, household education levels, household smoking status, childbearing age, birth weight, and alcohol intake. The cohort was further classified into two groups based on age: ≤ 12 years and 12–15 years. BMI was calculated as weight in kilograms divided by height in meters squared and divided into normal (BMI < 25 kg/m^2^), overweight (25 kg/m^2^ ≤ BMI < 30 kg/m^2^), and obesity (BMI ≥ 30 kg/m^2^). Race and ethnicity were categorized and recorded as Mexican American, other Hispanic, non-Hispanic White, non-Hispanic Black, and other races including other non-Hispanic; multiple non-Hispanic races were collected because they were considered together as a confounder. Family income was classified as the PIR and categorized into three levels (< 1.30, 1.30–3.49, or ≥ 3.50) (23). The health insurance status of the participants was divided as insurance covered or not insurance covered according to the responses to the questionnaire on health insurance. Household education levels were categorized as lower than high school, high school, college, and college graduate. Household smoking was divided as yes or no according the questionnaire of smoking-household smokers. Information about the participants’ early life included mother’s age at child’s birth (< 25, 25–34, and ≥ 35 years) and birth weight (low birth weight < 2500 g, and non-low birth weight ≥ 2500 g). Alcohol intake of the participants was divided as yes or no according the questionnaire on individual foods (first day).

### 2.4 Statistical analyses

Sample weights, strata, and primary sampling units were applied according to NHANES analytical guidelines (18). Weight was calculated based on the dietary day one sample weight divided by the number of cycles in the main analyses. Regarding data from the 2003–2004 to 2007–2008 cycle, dietary day one sample weight (WTDRD1) was used, and dietary day one 4-year sample weight (WTDR4YR) was used for data from the 1999–2000 to 2001–2002 cycle.

Data were presented as unweighted frequencies (weighted percentages) for categorical variables. Differences between groups were compared using the chi-squared test for categorical variables. Covariates with missing values were imputed using the multiple imputation by chained equations method. Using neurodevelopmental disorders as the dependent variable, batch univariate weighted logistic regression models with covariates as independent variables were constructed to examine influencing factors. The Generalized Variance Inflation Factor (GVIF) was used to assess multicollinearity in regression models that include both continuous and categorical predictor variables, and values exceeding 5 or 10 were considered problematic (24). The multifactorial weighted logistic regression models were utilized to compute the adjusted odds ratio (OR) and 95% confidence intervals (CI) for examining the associations between in-hospital NMS and the risk of neurodevelopmental disorders. Three models were constructed: Model 1 was a crude model that did not adjust for any covariate; Model 2 adjusted for age, sex, BMI, and race; and Model 3 adjusted for sex, age, BMI, race, PIR, household education levels, household smoking status, childbearing age, and birth weight.

We further stratified the analyses by covariates. The significance of the interactions was estimated using P-values for the interaction terms between in-hospital NMS and the stratified factors. We also conducted two sensitivity analyses. Sensitivity analysis 1: Weighted samples to reflect the target population were used, and non-weighted data were used to confirm the robustness of the weighted estimates. Sensitivity analysis 2: Imputed data help to reduce bias and to make the best use of available information; multiple interpolation was performed with the R package mice, and imputation stability was confirmed with non-imputed data.

All statistical analyses were performed from March 20 to April 26, 2024, using R software, version 4.3.0. A two-sided P < 0.05 was considered the threshold for statistical significance.

## 3 Results

### 3.1 Characteristics of the participants

Data from 15,502 participants were analyzed in this study (mean age, 8.05 years; participants age ≤12 years [weighted, 79.31%]; 7759 males [weighted, 50.99%]). Overall, 4970 (weighted, 13.33%) participants were Mexican American, 908 (weighted, 5.57%) were other Hispanic, 4372 (weighted, 60.21%) were non-Hispanic White, 4499 (weighted, 14.66%) were non-Hispanic Black, and 753 (6.24%) were categorized as other race. A total of 338 participants (weighted, 22.43%) with neurodevelopmental disorders and 1589 participants (weighted, 11.74%) who did not have neurodevelopmental disorders had experienced in-hospital NMS. Furthermore, 496 participants (weighted, 31.63%) with neurodevelopmental disorders and 2442 participants (weighted, 18.58%) without neurodevelopmental disorders had experienced family smoking. A total of 297 participants (weighted, 18.32%) with neurodevelopmental disorders had low birth weight while 1644 participants (weighted, 11.51%) without neurodevelopmental disorders had low birth weight. The baseline characteristics of the 15,502 participants are summarized according to their neurodevelopmental disorders and in-hospital NMS in **Table 1**.

**Table 1:** Baseline Characteristics of participants in NHANES 1999 to 2008^a^.

Variable	Total (n = 15502)	Neurodevelopmental disorders		*P*
		Yes (n=1652)	No (n=13850)	
In hospital NMS, n (%)				<.001
No	13575 (87.06)	1314 (77.57)	12261 (88.26)	
Yes	1927 (12.94)	338 (22.43)	1589 (11.74)	
Sex, n (%)				<.001
Male	7759 (50.99)	1067 (66.85)	6692 (48.97)	
Female	7743 (49.01)	585 (33.15)	7158 (51.03)	
Age, n (%)				<.001
≤12 years	11806 (79.31)	1084 (71.61)	10722 (80.29)	
>12 years	3696 (20.69)	568 (28.39)	3128 (19.71)	
BMI, n (%)				<.001
Normal	11911 (89.69)	1291 (85.58)	10620 (90.25)	
Overweight	1172 (6.85)	171 (8.72)	1001 (6.60)	
Obesity	694 (3.46)	132 (5.70)	562 (3.15)	
Race, n (%)				<.001
Mexican American	4970 (13.33)	387 (8.57)	4583 (13.93)	
Other Hispanic	908 (5.57)	91 (5.98)	817 (5.52)	
Non-Hispanic White	4372 (60.21)	529 (64.02)	3843 (59.72)	
Non-Hispanic Black	4499 (14.66)	582 (16.87)	3917 (14.38)	
Other Race^b^	753 (6.24)	63 (4.56)	690 (6.45)	
PIR, n (%)				<.001
<1.3	6501 (33.25)	741 (41.17)	5760 (32.23)	
1.3-3.49	5140 (37.22)	559 (38.49)	4581 (37.05)	
≥3.5	2838 (29.53)	256 (20.33)	2582 (30.72)	
Health insurance, n (%)				0.112
Not insured	2324 (11.23)	188 (9.23)	2136 (11.48)	
Insured	13053 (88.77)	1446 (90.77)	11607 (88.52)	
Household education, n (%)				<.001
<High school	5189 (22.19)	551 (25.79)	4638 (21.73)	
High School	3664 (25.06)	433 (27.87)	3231 (24.70)	
College	3773 (28.64)	424 (31.77)	3349 (28.24)	
College Graduate	2345 (24.11)	190 (14.57)	2155 (25.33)	
Household smoking, n (%)				<.001
No	12426 (79.94)	1143 (68.37)	11283 (81.42)	
Yes	2938 (20.06)	496 (31.63)	2442 (18.58)	
Childbearing age, n (%)				<.001
<25	6986 (38.01)	850 (47.93)	6136 (36.77)	
25-34	6900 (50.26)	622 (42.75)	6278 (51.20)	
≥35	1490 (11.73)	140 (9.32)	1350 (12.03)	
Low birth weight, n (%)				<.001
No (≥ 2500 g)	13145 (87.74)	1284 (81.68)	11861 (88.49)	
Yes (< 2500 g)	1941 (12.26)	297 (18.32)	1644 (11.51)	
Alcohol intake, n (%)				0.535
No	14818 (95.90)	1588 (96.39)	13230 (95.84)	
Yes	596 (4.10)	64 (3.61)	532 (4.16)	

a All estimates accounted for complex survey designs, and all percentages are weighted.

b Other race and ethnicity include other non-Hispanic, and non-Hispanic multiple races.

**Figure 2:**
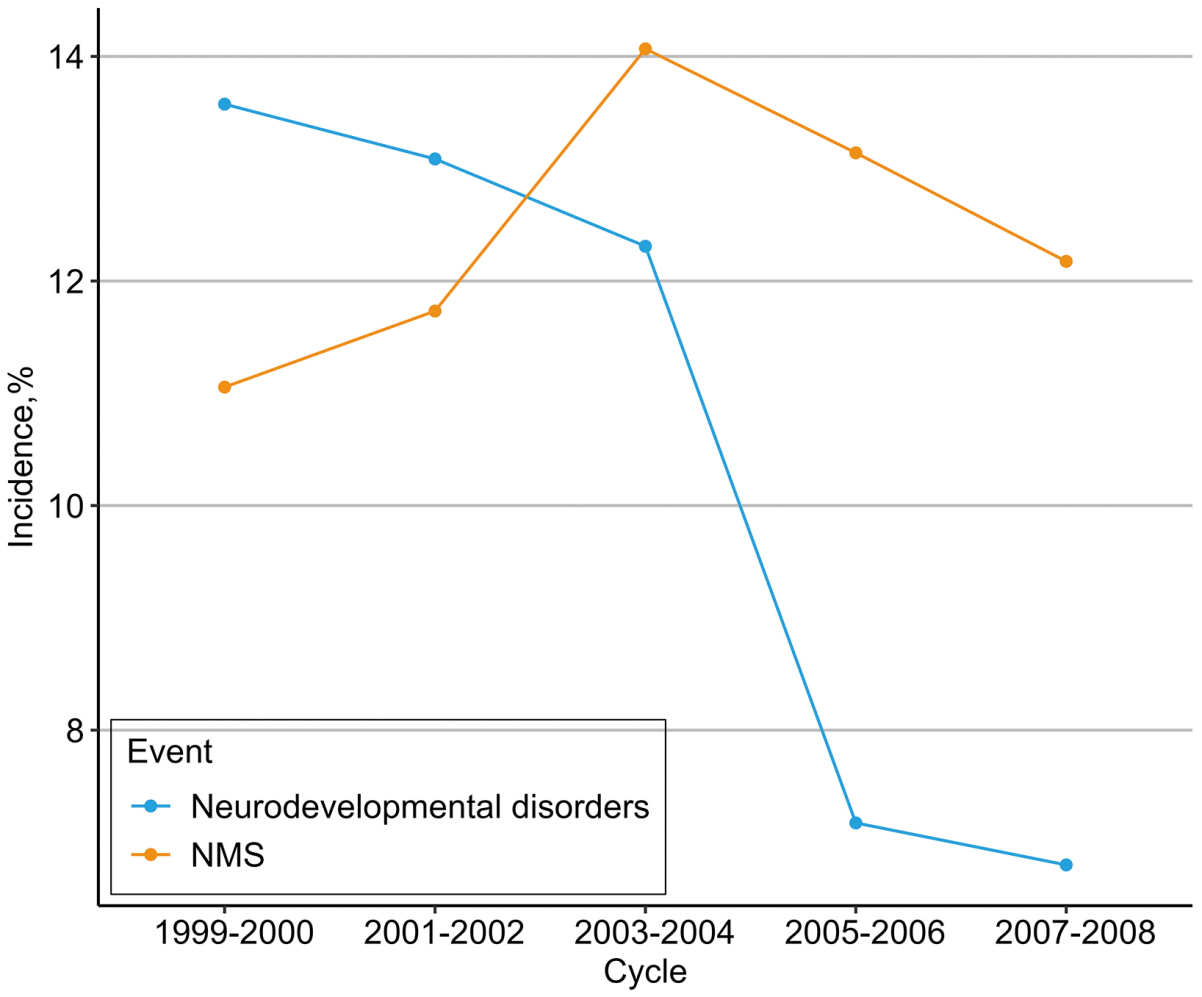
Incidence of in-hospital NMS and neurodevelopmental disorders. Cumulative incidence of neurodevelopmental disorders was 1927 (12.4%) for in-hospital NMS, and 1652 (10.7%) for neurodevelopmental disorders.

Within the analytical subjects, the incidence rate of hospitalized NMS showed a slight upward trend (11.06–12.18%) from the 1999–2000 to 2007–2008 survey cycle, while the incidence of neurodevelopmental disorders decreased significantly (13.58% to 6.80%). Additionally, 338 of the 1927 participants (17.54%) who were exposed to in-hospital NMS had a higher rate of neurodevelopmental disorders compared to 1314 of the 13,575 participants (9.68%) who were not exposed to NMS. The incidences of in-hospital NMS and neurodevelopmental disorders sorted according to survey cycles are shown in **Figure 2**.

### 3.2 Association between in-hospital NMS and neurodevelopmental disorders

Univariate weighted regression analyses revealed that covariates such as sex, age, BMI, race, PIR, household education levels, household smoking status, childbearing age, and birth weight had an impact on neurodevelopmental disorders (**Supplementary Table S1**). In our analysis, the largest GVIF ranged from 1 to 2, indicating negligible variance inflation, confirming that the multivariate regression model was not biased by multicollinearity (**Supplementary Table S2**).

Subsequently, we employed a multi-model, multi-factor weighted logistic regression analysis strategy to examine the correlation. In the crude model, the OR for neurodevelopmental disorders was 2.18 (95% CI, 1.75–2.71) compared with the reference group (P < 0.001). Accounting for sex, age, BMI, and race, the adjusted OR was 2.03 (95% CI, 1.61–2.56) compared with the reference group (P < 0.001). After multivariable adjustment in Model 3, there was still a significant association between in-hospital NMS and neurodevelopmental disorders; the adjusted OR was 1.82 (95% CI, 1.40–2.37) compared with the reference group (P <0 .001) (**Table 2**).

**Table 2:** Associations between in-hospital NMS and neurodevelopmental disorders in NHANES, 1999 to 2008.

Variable	Model 1^a^		Model 2^b^		Model 3^c^	
	OR (95% CI)	P value	OR (95% CI)	P value	OR (95% CI)	P value
In-hospital NMS						
No	1.00 (Reference)		1.00 (Reference)		1.00 (Reference)	
Yes	2.18(1.75-2.71)	<0.001	2.028(1.61-2.56)	<0.001	1.82(1.40-2.37)	<0.001

a Crude model.

b Adjusted for age, sex, BMI and race.

c Further adjusted for PIR, household education levels, household smoking status, childbearing age and birth weight.

### 3.3 Stratified and sensitivity analyses

**Figure 3:**
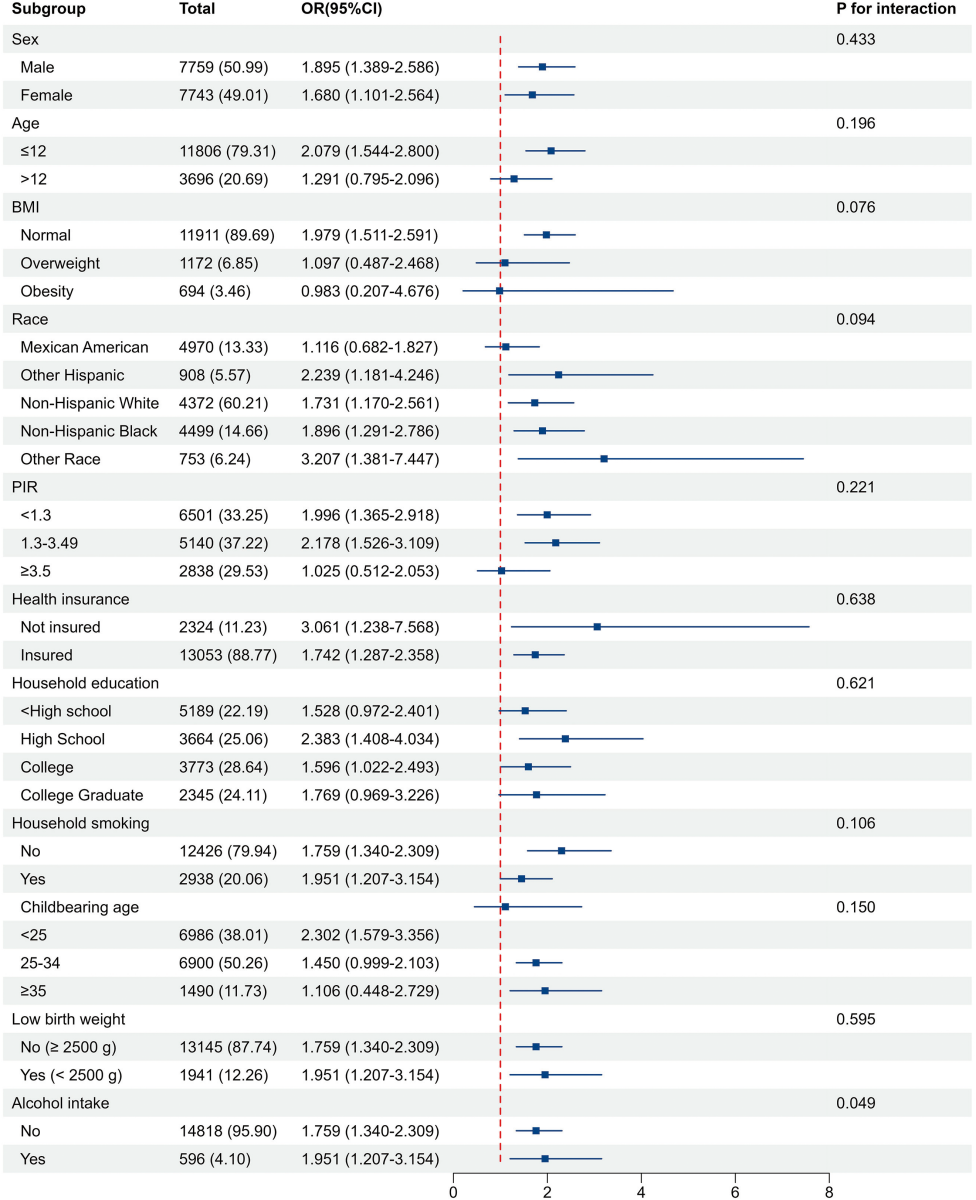
Associations of in-hospital NMS with neurodevelopmental disorder in subgroups. The model was adjusted for age, sex, BMI, race, PIR, health insurance status, household education levels, household smoking status, childbearing age, birth weight, and alcohol intake. The strata variable was not included in the adjustment when stratifying by itself.

Subgroup analyses indicated that exposure to in-hospital NMS was associated with a continuous increase in the risk of neurodevelopmental disorders in the subgroups (**Figure 3**). The study populations were stratified according to age (>12), BMI (overweight, obesity), race (Mexican American, other Hispanic, non-Hispanic white, non-Hispanic black, other race), household education level (< high school, college, college graduate), and childbearing age (25–34, ≥ 35 years), this association was not statistically significant (P > 0.05) (**Supplementary Table S3**). Interaction analyses revealed that the interactions between each covariate and in-hospital NMS had no significant impact on the relationship between in-hospital NMS and neurodevelopmental disorders except alcohol intake (P for Interaction = 0.049). Two sensitivity analyses revealed no substantial changes in these findings (**Supplementary Table S4-5**).

## 4 Discussion

This is the first large-scale cross-sectional study to examine the long-term effect of in-hospital NMS on neurodevelopmental disorders in children and adolescents. Our results showed a clear downward trend in the incidence rate of neurodevelopmental disorders, while in-hospital NMS showed a slightly fluctuating increase in the 1999–2000 to 2007–2008 survey cycle. Ultimately, we found that in-hospital NMS was associated with a higher risk of neurodevelopmental disorders compared to non-NMS, after controlling for multiple confounding factors. Stratified analyses and sensitivity analyses demonstrated the stability and reliability of our results.

In contemporary society, most women choose to give birth in a hospital, regardless of whether they have a cesarean section or a natural birth, and this has significantly increased the survival rate of newborns (25). Early social experiences have a profound long-term impact on development outcomes as they occur when the brain is best able to be permanently programmed due to the exceptional brain plasticity during this period (26). Studies on maternal separation have shown that the neuropsychological status of mothers changes after the separation, and these mental changes would also affect the neurological development of the offspring (27). Studies have also shown early mother-infant contact is associated with several benefits, including promoting breastfeeding, fostering emotional attachment, improving physiological stability, reducing crying, promoting sleep, and supporting neurodevelopment (4, 28). This is the first large-scale cross-sectional study on the role of in-hospital NMS on neurodevelopmental disorders in children and adolescents that used data from a nationwide scale. After adjusting for confounders, weighted logistic regression analysis revealed a significant positive correlation between in-hospital NMS and neurodevelopmental disorders, with an adjusted OR of 1.82 (95% CI 1.40–2.37, P < 0.001). Therefore, it is critical to utilize a large longitudinal cohort study to further investigate the potential contributing factors and long-term consequences of in-hospital NMS and neurodevelopmental disorders.

In this study, we identified possible demographic and preterm neonatal influencing factors as covariates based on previous research and theoretical hypotheses combined with data collected from the NHANES database. Although some subgroup analyses showed no statistically significant differences (**Supplementary Table S3**), the overall risk of neurodevelopmental disorders continued to increase in all subgroups. Furthermore, interaction analyses revealed no subgroup effects for each covariate except alcohol intake. Studies have also confirmed that alcohol influences the development of the central nervous system and may lead to neurodevelopmental disorders (29). Subgroup stratification by alcohol intake may serve as a basis for screening high-risk populations or patients. Existing evidence suggests that in-hospital NMS may be considered a major cause of neurodevelopmental disorders in children and adolescents. Moreover, previous evidence suggests that NMS may affect several aspects of maternal and newborn health (10–13, 27) and could serve as a comprehensive assessment indicator that is closely correlated with neurodevelopmental disorders. Although the average follow-up time in this study is 8.05 years from birth, future studies should have a longer follow-up period to facilitate further investigation of potential confounding factors in children and adolescents.

Another notable result of this study was that the incidence of in-hospital NMS showed a slightly fluctuating increase, while the incidence of neurodevelopmental disorders showed a clear downward trend. Previous studies have shown that newborns in the United States are increasingly likely to be admitted to neonatal intensive care units each year, and this increases the possibility of neonatal intensive care units overuse (30, 31). Additionally, studies have shown that family-integrated care and kangaroo mother care can improve chronic physiological stress in mothers of premature infants and influence children’s behaviors (15, 16). This provides a reference for improving the outcome of in-hospital NMS. Future rigorously designed prospective longitudinal cohort studies are needed to demonstrate the association between care model and in-hospital NMS outcomes in children and adolescents, not just only preterm infants. Our results may also provide certain guidelines for the treatment of in-hospital NMS and the design and care model of special neonatal care facilities, aiming to improve the long-term prognosis caused by in-hospital NMS.

### 4.1 Strengths and limitations

We used nationally representative data from NHANES, which allowed us to generalize our results to a broader population. Existing studies on NMS mainly focused on preschool age under 5 years of age (10, 32, 33); however, as the average follow-up period in this study was 8.05 years, it better reflected the long-term effects of in-hospital NMS in children and adolescents. The extensive follow-up period facilitated validation of both in-hospital NMS and neurodevelopmental disorders within a nationally representative sample and enabled examination of the association in children and adolescents.

This study has some limitations. First, a previous study reported that admitting preterm infants to neonatal intensive care units can influence their neurological development (34); however, this study lacked data on potential confounders, including preexisting congenital comorbidities, surgical or pharmacological interventions (e.g., anesthesia, medication exposure), and gestational age at birth. Second, this study could not determine the duration of in-hospital NMS and excluded instances where mother-child separation occurred at a different stage of life. Third, neonatal separation data were based on self-reports, which were subject to recall bias and may consequently lead to an underestimation of its incidence. Finally, due to the extensive rate of missing data in the NHANES dataset, particularly regarding breastfeeding status and maternal smoking during pregnancy, it was not feasible to analyze these covariables, which may have introduced potential confounding factors that influenced the study outcome (35, 36). Furthermore, some potential confounders may have not been adequately accounted for, leaving residual and unidentified confounders that cannot be completely ruled out.

## 5 Conclusions

Overall, our study is the first to examine the association between in hospital NMS and the risk of neurodevelopmental disorders in children and adolescents using cross-section data from a nationally representative cohort of children and adolescents. Our findings support the hypothesis that in-hospital NMS is a risk factor for neurodevelopmental disorders in children and adolescents. Although our results are preliminary, they support the evaluation of interventions targeting in-hospital NMS, thereby changing the care model or inpatient ward design.

## Data Availability

Publicly available datasets were analyzed in this study. This data can be found here: The National Health and Nutrition Examination Survey (NHANES) data are publicly available at https://www.cdc.gov/nchs/nhanes/ (accessed June 18, 2025).
