# Supplementary material for: In-hospital neonatal maternal separation as early-life stress and neurodevelopmental disorders: a cross-sectional and population-based study
Source: Front Psychiatry. 2025 Jun 27;16:1572600. doi: 10.3389/fpsyt.2025.1572600 (PMC12245852; doi:10.3389/fpsyt.2025.1572600)
Supplement: Supplementary file 1 [file DataSheet1.docx]

**DATA SUPPLEMENT**

**In-hospital neonatal maternal separation as early-life stress and neurodevelopmental disorders: A cross-sectional and population-based study**

**List of Supplemental Materials**

**Table S1**. Batch univariate logistic regression results

**Table S2**. VIF for all variables.

**Table S3**. Stratified analyzes between in-hospital NMS and neurodevelopmental disorders

**Table S4.** Associations of in-hospital NMS and neurodevelopmental with unweighted data

**Table S5**. Associations of in-hospital NMS and neurodevelopmental with non-imputed data

**Table S1. Batch univariate logistic regression results**

| Variables | OR (95%CI) | P Value |
| --- | --- | --- |
| Sex |  |  |
| Male | Reference |  |
| Female | 0.48 (0.40-0.57) | <0.001 |
| Age |  |  |
| ≤12 | Reference |  |
| >12 | 1.62 (1.29-2.02) | <0.001 |
| BMI |  |  |
| Normal | Reference |  |
| Overweight | 1.39 (1.11-1.74) | 0.005 |
| Obesity | 1.91 (1.31-2.78) | 0.001 |
| Race |  |  |
| Mexican American | Reference |  |
| Other Hispanic | 1.76 (1.12-2.76) | 0.017 |
| Non-Hispanic White | 1.74 (1.41-2.15) | <0.001 |
| Non-Hispanic Black | 1.91 (1.50-2.42) | <0.001 |
| Other Race | 1.15 (0.73-1.80) | 0.55 |
| PIR |  |  |
| <1.3 | Reference |  |
| 1.3-3.49 | 0.81 (0.65-1.02) | 0.072 |
| ≥3.5 | 0.52 (0.42-0.64) | <0.001 |
| Health insurance |  |  |
| Not insured | Reference |  |
| Insured | 1.28 (0.94-1.73) | 0.12 |
| Household education |  |  |
| <High school | Reference |  |
| High School | 0.95 (0.77-1.17) | 0.63 |
| College | 0.95 (0.77-1.17) | 0.61 |
| College Graduate | 0.49 (0.36-0.65) | <0.001 |
| Household smoking |  |  |
| No | Reference |  |
| Yes | 2.03 (1.67-2.46) | <0.001 |
| Childbearing age |  |  |
| <25 | Reference |  |
| 25-34 | 0.64 (0.54-0.76) | <0.001 |
| ≥35 | 0.59 (0.41-0.86) | 0.008 |
| Low birth weight |  |  |
| No (≥ 2500 g) | Reference |  |
| Yes (< 2500 g) | 1.73 (1.36-2.20) | <0.001 |
| Alcohol intake |  |  |
| No | Reference |  |
| Yes | 0.86 (0.54-1.38) | 0.54 |

PIR = Poverty Income Ratio.

**Table S2. GVIF for all variables**

| **variable** | **GVIF** |
| --- | --- |
| In-hospital NMS | 1.07 |
| Sex | 1.00 |
| Age | 1.05 |
| BMI | 1.02 |
| Race | 1.04 |
| PIR | 1.11 |
| Health insurance | 1.05 |
| Household Education | 1.09 |
| Household smoking | 1.06 |
| Childbearing age | 1.03 |
| Low birth weight | 1.08 |
| Alcohol intake | 1.00 |

GVIF = Generalized Variance Inflation Factor. NMS = Neonatal Maternal Separation. PIR = Poverty Income Ratio.

**Table S3. Stratified analyzes between in-hospital NMS and neurodevelopmental disorders**

| Variables | OR (95%CI) | P Value | P for Interaction |
| --- | --- | --- | --- |
| Sex |  |  | 0.43 |
| Male | 1.91 (1.39-2.60) | <0.001 |  |
| Female | 1.67 (1.09-2.58) | 0.025 |  |
| Age |  |  | 0.20 |
| ≤12 | 2.09 (1.55-2.81) | <0.001 |  |
| >12 | 1.29 (0.79-2.11) | 0.32 |  |
| BMI |  |  | 0.076 |
| Normal | 1.99 (1.52-2.61) | <0.001 |  |
| Overweight | 1.09 (0.48-2.44) | 0.84 |  |
| Obesity | 0.98 (0.21-4.64) | 0.98 |  |
| Race |  |  | 0.094 |
| Mexican American | 1.12 (0.68-1.83) | 0.79 |  |
| Other Hispanic | 2.10 (1.09-4.03) | 0.43 |  |
| Non-Hispanic White | 1.74 (1.17-2.57) | 0.38 |  |
| Non-Hispanic Black | 1.90 (1.30-2.78) | 0.35 |  |
| Other Race | 3.38 (1.44-7.89) | 0.38 |  |
| PIR |  |  | 0.22 |
| <1.3 | 2.00 (1.37-2.92) | 0.002 |  |
| 1.3-3.49 | 2.18 (1.53-3.11) | <0.001 |  |
| ≥3.5 | 1.03 (0.51-2.05) | 0.94 |  |
| Health insurance |  |  | 0.64 |
| Not insured | 3.07 (1.24-7.63) | 0.021 |  |
| Insured | 1.75 (1.29-2.37) | <0.001 |  |
| Household education |  |  | 0.62 |
| <High school | 1.50 (0.96-2.33) | 0.10 |  |
| High School | 2.39 (1.40-4.06) | 0.009 |  |
| College | 1.60 (1.02-2.50) | 0.067 |  |
| College Graduate | 1.80 (0.98-3.31) | 0.086 |  |
| Household smoking |  |  | 0.11 |
| No | 2.29 (1.57-3.33) | <0.001 |  |
| Yes | 1.47 (1.01-2.14) | 0.055 |  |
| Childbearing age | 1.12 (0.46-2.73) | 0.80 |  |
| <25 |  |  | 0.15 |
| 25-34 | 1.64 (1.23-2.19) | 0.002 |  |
| ≥35 | 2.31 (1.45-3.67) | 0.001 |  |
| Low birth weight |  |  | 0.60 |
| No (≥ 2500 g) | 1.76 (1.34-2.32) | <0.001 |  |
| Yes (< 2500 g) | 4.53 (1.87-10.99) | 0.002 |  |
| Alcohol intake |  |  | 0.049 |
| No | 1.76 (1.33-2.33) | <0.001 |  |
| Yes | 2.00 (1.23-3.25) | 0.008 |  |

PIR = Poverty Income Ratio.

**Table S4. Associations of in-hospital NMS and neurodevelopmental with unweighted data**

|  | Model 1 ^a^ | |  | Model 2 ^b^ | |  | Model 3 ^c^ | | |
| --- | --- | --- | --- | --- | --- | --- | --- | --- | --- |
|  | OR (95% CI) | P value |  | OR (95% CI) | P value |  | OR (95% CI) | | P value |
| In-hospital NMS |  |  |  |  |  |  |  |  | |
| No | 1.00 (Reference) |  |  | 1.00 (Reference) |  |  | 1.00 (Reference) |  | |
| Yes | 1.99 (1.74-2.26) | <0.001 |  | 1.94 (1.70-2.22) | <0.001 |  | 1.74 (1.50-2.01) | <0.001 | |

NMS = Neonatal Maternal Separation. PIR = Poverty Income Ratio.

^a^ Crude model.

^b^ Adjusted for age, sex, BMI and race.

^c^ Further adjusted for PIR, household education levels, household smoking status, childbearing age and birth weight.

**Table S5. Associations of in-hospital NMS and neurodevelopmental with non-imputed data**

|  | Model 1 ^a^ | |  | Model 2 ^b^ | |  | Model 3 ^c^ | |
| --- | --- | --- | --- | --- | --- | --- | --- | --- |
|  | OR (95% CI) | P value |  | OR (95% CI) | P value |  | OR (95% CI) | P value |
| In-hospital NMS |  |  |  |  |  |  |  |  |
| No | 1.00 (Reference) |  |  | 1.00 (Reference) |  |  | 1.00 (Reference) |  |
| Yes | 1.99 (1.74-2.26) | <0.001 |  | 1.84 (1.60-2.11) | <0.001 |  | 1.63 (1.38-1.92) | <0.001 |

NMS = Neonatal Maternal Separation. PIR = Poverty Income Ratio.

^a^ Crude model.

^b^ Adjusted for age, sex, BMI and race.

^c^ Further adjusted for PIR, household education levels, household smoking status, childbearing age and birth weight.
